# Immunomodulatory Activity of Mesenchymal Stem Cells in Lupus Nephritis: Advances and Applications

**DOI:** 10.3389/fimmu.2022.843192

**Published:** 2022-03-10

**Authors:** Jicui Li, Manyu Luo, Bing Li, Yan Lou, Yuexin Zhu, Xue Bai, Baichao Sun, Xuehong Lu, Ping Luo

**Affiliations:** Department of Nephrology, The Second Hospital of Jilin University, Changchun, China

**Keywords:** mesenchymal stem cells, lupus nephritis, immunomodulation, immune cells, cytokines, autoimmune

## Abstract

Lupus nephritis (LN) is a significant cause of various acute and chronic renal diseases, which can eventually lead to end-stage renal disease. The pathogenic mechanisms of LN are characterized by abnormal activation of the immune responses, increased cytokine production, and dysregulation of inflammatory signaling pathways. LN treatment is an important issue in the prevention and treatment of systemic lupus erythematosus. Mesenchymal stem cells (MSCs) have the advantages of immunomodulation, anti-inflammation, and anti-proliferation. These unique properties make MSCs a strong candidate for cell therapy of autoimmune diseases. MSCs can suppress the proliferation of innate and adaptive immune cells, such as natural killer cells (NKs), dendritic cells (DCs), T cells, and B cells. Furthermore, MSCs suppress the functions of various immune cells, such as the cytotoxicity of T cells and NKs, maturation and antibody secretion of B cells, maturation and antigen presentation of DCs, and inhibition of cytokine secretion, such as interleukins (ILs), tumor necrosis factor (TNF), and interferons (IFNs) by a variety of immune cells. MSCs can exert immunomodulatory effects in LN through these immune functions to suppress autoimmunity, improve renal pathology, and restore kidney function in lupus mice and LN patients. Herein, we review the role of immune cells and cytokines in the pathogenesis of LN and the mechanisms involved, as well as the progress of research on the immunomodulatory role of MSCs in LN.

## Introduction

Systemic lupus erythematosus (SLE) is a chronic autoimmune inflammatory disease with manifestations of multi-organ damage due to extensive deposition of immune complexes (IC). Kidney involvement in SLE is lupus nephritis (LN), and approximately 20–60% of SLE cases eventually develop LN (1) and is the main predictor of poor prognosis in SLE. The development of LN is based on the loss of immune tolerance to self-nuclear antigens and inflammation induced by the IC. Deposition of antigen-antibody complexes in the kidney induces LN, which triggers an inflammatory cascade that includes complement activation, activation of Fc receptors, activation of renal lamina propria cells and aggregation of inflammatory cells, and further activation of the immune system by mediators released by tissue injury, T cells, B cells, dendritic cells (DCs), macrophages, and the cytokines they produce. This can ultimately lead to LN by disrupting immune tolerance and inducing the onset of inflammation (2, 3). Despite significant advances in the diagnosis and treatment of SLE, its prevalence has increased over time (4) In addition, the treatment of refractory LN has become a subject of great interest as some refractory patients do not achieve the expected efficacy with standard therapies (hormones and immunosuppressants) and experience considerable side effects (infections and secondary malignancies).

Mesenchymal stem cells (MSCs) are pluripotent stem cells with abundant sources, such as bone marrow, umbilical cord, umbilical blood, adipose tissue, and embryonic tissue (5). MSCs have been shown to have immunomodulatory abilities and reduce inflammatory responses (6). Mesenchymal stem cell transplantation (MSCT) has been used to treat a variety of autoimmune diseases, including SLE, and has benefited patients who are resistant to conventional therapies (7, 8). In recent years, research related to stem cell therapy for LN has been rapidly developing. In this review, we focus on the role of various immune cells and cytokines in LN and how MSCs exert immunomodulatory activity in LN by regulating the corresponding cells or cytokines, which provides a reference for MSCs to target and regulate specific cells and cytokines.

## Defective MSCs in SLE

One research study found that there was no treatment effect within 14 days after autologous MSCT in two SLE patients (9). Other numerous reports have suggested that MSCs in SLE are defective and may be involved in the pathogenesis of SLE, and therefore, SLE is assumed to be a “stem cell disease”. MSCs of SLE patients grow more slowly than those of healthy individuals and have a lower proliferative capacity; normal MSCs are uniformly shuttle-shaped, but MSCs of SLE patients become bulky and flattened from the third generation onwards (10). MSCs from SLE patients show signs of senescence such as deep staining of nuclei, disrupted F-actin cytoskeleton, increased reactive oxygen species production, increased senescence associated β-galactosidase staining, increased telomerase activity, and increased DNA damage and repair (10–12). The endoplasmic reticulum stress response induces G1 cell cycle arrest in bone marrow MSCs of SLE patients and is involved in the senescence of MSCs in SLE patients (13). The mitochondrial antiviral signaling protein (MAVS) is an articulatory protein that induces IFN-β, and the MAVS-IFNβ-positive feedback loop mediates the senescence of MSCs in SLE (12). Studies have shown that aberrant activation of several signaling pathways, such as Wnt/β-catenin (14), p53/p21 (14), PI3K/Akt (15), PTEN/Akt-p27 (16), and JAK-STAT (11), is involved in the aging process of MSCs in SLE. At the same time, MSCs in SLE exhibit impaired migration, differentiation, and immunomodulation (17, 18). Expression levels of mRNA for IL-6 and IL-17 are downregulated in MSCs from SLE patients (10). Indoleamine 2,3 dioxygenase (IDO) is a tryptophan metabolizing enzyme. The mRNA level of IDO secreted by bone marrow MSCs from patients with active lupus was significantly reduced, affecting its suppressive effect on T cells (19). Decreased CCL2 expression in bone marrow MSCs of SLE affects their inhibitory effect on B cells (17). These findings suggest that MSCs from SLE are defective.

## Immunomodulatory Activity of MSCs on Immune Cell Subsets in LN

**Figure 1:**
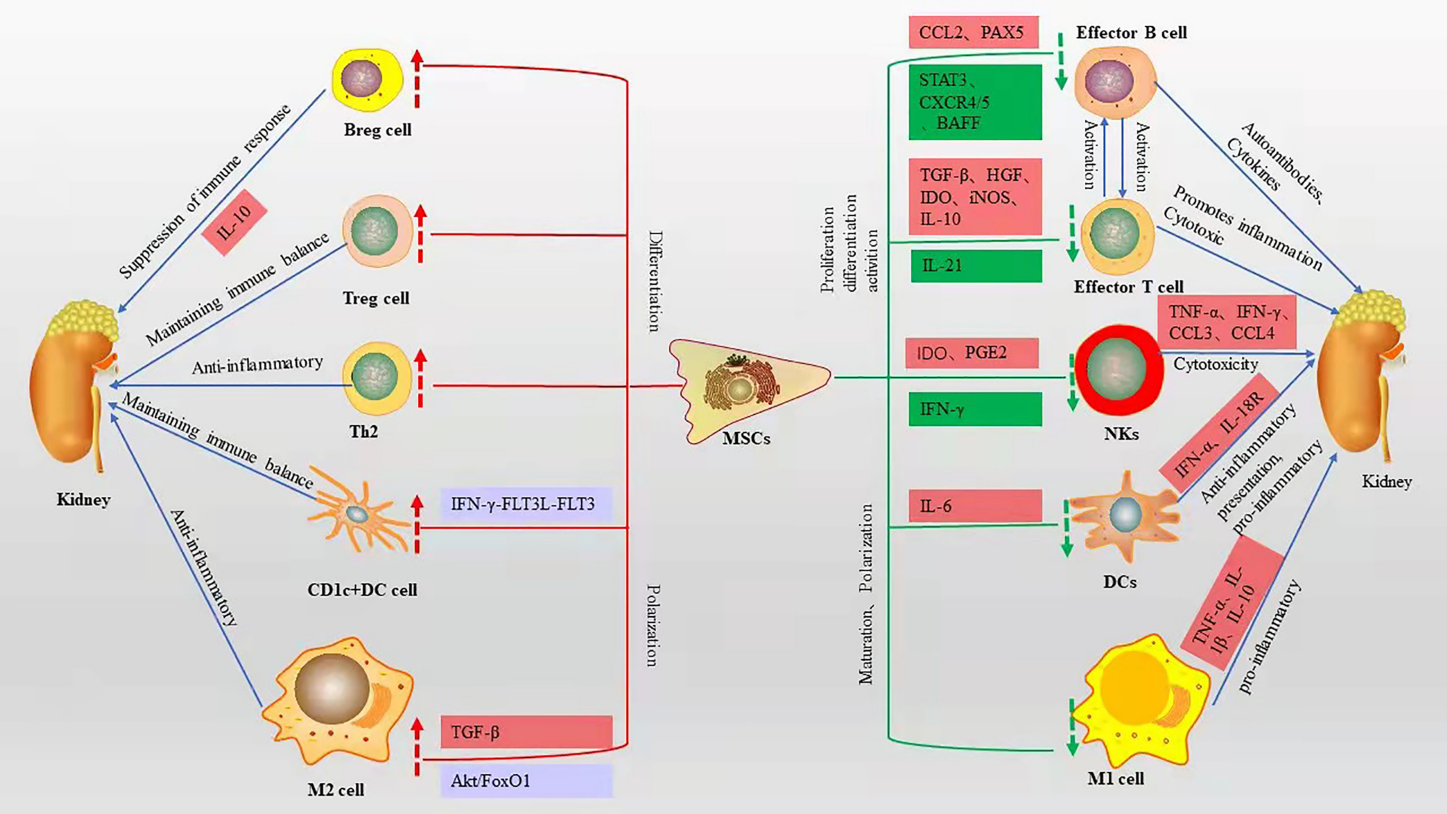
Immunomodulatory effects of MSCs on various immune cells and related mechanisms, red boxes represent elevated, green boxes represent decreased, blue boxes represent pathways or signaling axes. The red up arrow represents an upward adjustment and the green down arrow a downward adjustment.

SLE is characterized by T-cell dysfunction and polyclonal B-cell activation, and various immune cells and cytokines are involved in the pathogenesis of LN (20). Owing to their unique immunomodulatory properties (21), MSCs are strong candidates for cell therapy and can be used in a wide range of immune-related diseases. MSCs can polarize various immune cells to an inactive or anti-inflammatory state (22), while inhibiting their proliferation and attenuating cytotoxicity (**Figure 1**).

### Immunomodulatory Activity of MSCs on B Cells

B cells are a hallmark of the adaptive immune system. Researchers have shown lupus-prone mice lacking B cells do not develop nephritis (23). In contrast, lupus-like renal disease occurs in wild-type mice injected with lupus-derived autoantibodies (24). B cells can produce autoantibodies against nuclear proteins and DNA, especially anti-double-stranded DNA (anti-dsDNA) (25). Meanwhile, B cells can act as antigen-presenting cells and promote the activation of T cells in MRL-lpr/lpr mice (26), and different subpopulations of B cells produce large amounts of cytokines involved in immune regulation (27). Regulatory B cells (Breg) are a class of B cells with immunomodulatory functions that play an important role in maintaining immune tolerance and suppressing harmful immune responses (28). Plasma cells are effector B cells and are present in large numbers in the tubulointerstitium of SLE-prone NZB/W mice, and the degree of infiltration correlates positively with ds-DNA-IgG titer and the severity of LN pathology (29). B cell activating factor (BAFF) is a TNF family cytokine secreted mainly by DCs, macrophages, and neutrophils, and the development and survival of B cells depend on BAFF stimulation (30). CD4+ and CD8+ T cells from SLE patients express BAFF mRNA, but not in normal subjects (31). Severe proteinuria and mortality were shown to be significantly reduced in BAFF-deficient 6–7-month-old NEM mice (32), in addition, BAFF transgenic (Tg) mice showed a large increase in the number of mature B cells and effector T cells, similar to human SLE, anti-dsDNA antibodies, and immunoglobulin deposits in the kidney (33).

**Figure 2:**
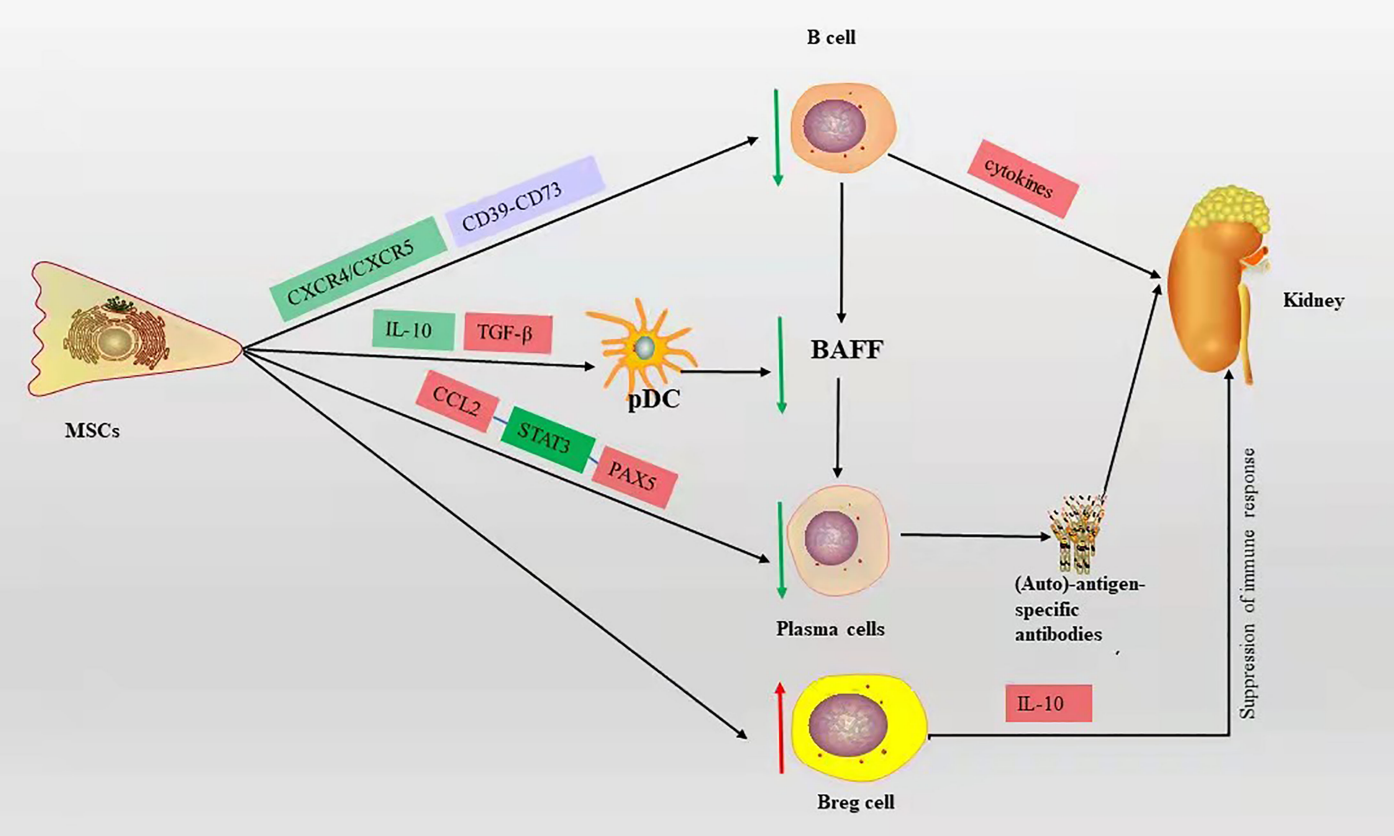
The mechanism of B cell action on LN and the immunomodulatory mechanism of MSCs on B cells in LN, red box represents elevation, green box represents decrease, blue box represents pathway or signaling axis. The red up arrow represents an upward adjustment and the green down arrow a downward adjustment.

MSCs can affect the proliferation and differentiation of B cells, reduce plasma cell production, decrease the secretion of immunoglobulins, and inhibit B cell chemotaxis (34). The MSC-derived chemokine ligand 2 (CCL2) inhibits plasma cell production of immunoglobulins by suppressing activation of transcription factor 3 (STAT3) and inducing transcription factor PAX5 (17, 35). MSCs can downregulate chemokine receptor 4/5(CXCR4/CXCR5) chemotactic receptors, thereby inhibiting B cell chemotaxis (36). Ma et al. (37) showed that bone marrow MSCs derived from BALB/c mice injected intravenously into MRL/lpr mice inhibited B cell maturation and differentiation by suppressing BAFF production, resulting in a significant reduction in proteinuria and glomerular inflammation. Meanwhile, *in vitro* experiments showed that bone marrow MSCs from BALB/c mice may inhibit the decrease in BAFF levels secreted by DCs through downregulation of IL-10 and upregulation of transforming growth factor (TGF-β), leading to B cell activation and immunoglobulin production. Transplantation of human-derived adipose MSCs into Roquin ^san/san^ C57BL/6 mice *via* tail vein increased IL-10-producing Breg cells and significantly improved kidney tract proliferation and interstitial inflammatory cell infiltration, as well as increased IL-10-producing Breg cells *in vitro* (38). Human gingival-derived MSCs inhibit B cell proliferation, activation, plasma cell differentiation, and improve LN symptoms such as reduced proteinuria and glomerulonephritis, which through the CD39-CD73 signaling axis *in vitro* and *in vivo* (39). These studies suggest that MSCs can improve LN symptoms by inhibiting the proliferation, differentiation, and chemotaxis of B cells (**Figure 2**).

### Immunomodulatory Activity of MSCs on T Cells

**Figure 3:**
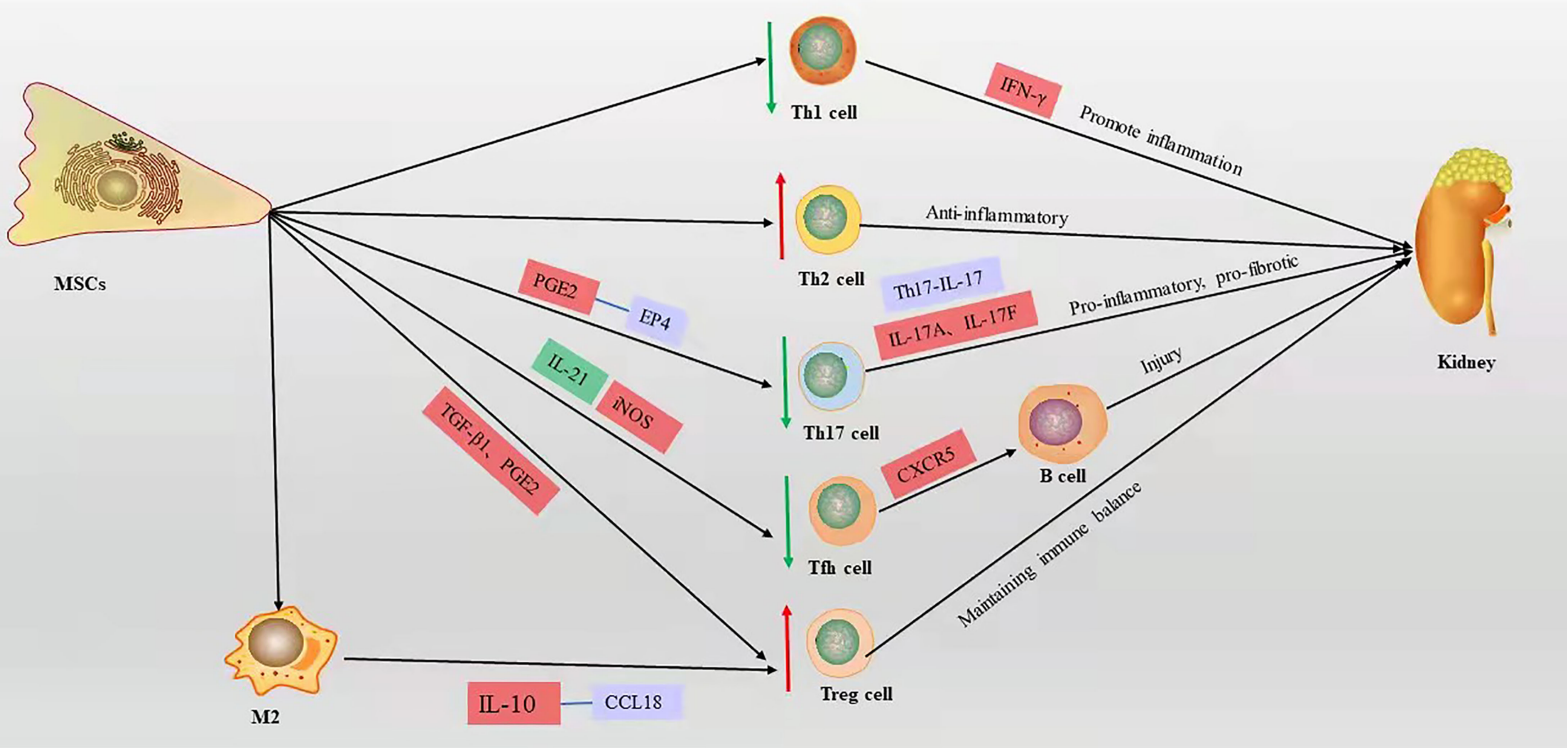
The mechanism of T cell action on LN and the immunomodulatory mechanism of MSCs on T cells in LN, red box represents elevation, green box represents decrease, blue box represents pathway or signaling axis. The red up arrow represents an upward adjustment and the green down arrow a downward adjustment.

T cells play a central and multiple role in LN and are another type of adaptive immune cell. T cells amplify the inflammatory response by secreting various proinflammatory cytokine cascades and assist B cells to produce autoantibodies. Imbalance of T cell subsets can affect the course of LN. Large numbers of T cells infiltrate the LN kidney tissue through cytotoxicity or by promoting the activation and recruitment of macrophages and NKs, which directly or indirectly damage renal parenchymal cells (40). Many functional defects of CD4+ T cells in SLE patients (41, 42). Activated CD4+ T cells can differentiate into various T helper (Th) subpopulations with different functions, such as Th1, Th2, Th17, follicular helper T (Tfh) cells, and regulatory T (Treg) cells (43, 44). TGF-β and hepatocyte growth factor (HGF) mediate the inhibition of T cell proliferation by MSCs, leading to a decrease in cyclin D2 and an increase in p27 kip1z at the molecular level, resulting in stagnation of T cell proliferation in G1 phase (45, 46). Plumas et al. (47) showed that the conversion of tryptophan to kynurenine by MSC-derived IDO in the presence of IFN-γ induces apoptosis in activated T cells. Bone marrow MSCs mediate T cell apoptosis *via* the FAS ligand/FAS pathway in the systemic sclerosis disease model (48). However, the lack of functional Fas in B6.lpr mice leads to a defect in the T cell apoptotic process, and it has been shown that umbilical cord MSCs promote apoptosis of CD4+ T cells in B6.lpr mice and have a significant effect in an *in vitro* setting, the mechanism of which is not yet clear (49). MSCs induce a shift from a proinflammatory to an anti-inflammatory state in T cells (50). Collectively, these reports show that MSCs improve LN by suppressing T cell proliferation, promoting apoptosis, and improving inflammatory status (**Figure 3**).

## Immunomodulatory Activity of MSCs on Th1/Th2

An imbalance in T cell subsets severely affects the disease process in LN. Elevated Th1/Th2 (IFN-γ/IL-4) ratios in peripheral blood is a characteristic feature of LN, and the balance of Th1/Th2 in peripheral cells of LN patients shows a polarization toward the Th1 phenotype, and the expression level of IFN-γ produced by Th1 cells parallels the severity of renal injury (51). Sigdel et al. (52) showed that Th1 phenotype cells predominate in peripheral blood in SLE with diffuse proliferative LN. As in human diffuse proliferative LN, MRL/lpr mouse nephritis was associated with a polarized Th1 cell phenotype (53). Allogeneic chondrogenic MSCs suppress the differentiation and proliferation of pro-inflammatory Th1 cells and promote the differentiation of T cells into the anti-inflammatory Th2 subpopulation (54). Choi et al. (55) demonstrated that long-term continuous administration of human adipose tissue-derived MSCs downregulated the Th1/Th2 ratio in the spleen of C3.MRL-Fas ^lpr^/J mice, significantly reduced anti-dsDNA levels, and decreased renal inflammatory cell infiltration and glomerular C3 deposition. Human umbilical cord-derived MSCs reduced the Th1/Th2 ratio in the spleen of B6.lpr mice, improved renal function, and reduced infiltration of renal lesions and inflammatory cells (49). Thus, MSCs improve LN by balancing the Th1/Th2 ratio.

## Immunomodulatory Activity of MSCs on Th17/Treg

Sakaguchi et al. (56) isolated a unique type of CD4+ CD25+ T cells capable of suppressing immune responses and maintaining immune tolerance, which were later named Treg cells. The presence of a limited number and/or impaired function of Treg cells in patients with SLE is associated with increased disease activity (57, 58). The defective immune response of Treg cells in SLE is characterized by reduced expression of CD4, CD25, and Forkhead box P3 (Foxp3), known as inducible Treg (iTreg) (59). Foxp3 is a transcription factor that is important for Treg cell development, function, and maintenance of immune homeostasis (60, 61). IL-17A and IL-17F are the major cytokines secreted by Th17 cells (62), and their receptors are expressed in most renal cells and are involved in activating pro-inflammatory and pro-fibrotic pathways. Th17 is involved in the pathogenesis of LN (63, 64); Th17/IL-17 axially recruits Th17 cells in the kidney and is involved in pro-inflammatory responses and pro-fibrotic actions in the kidney, leading to renal fibrosis and loss of renal function (65). The increase in peripheral Th17 cells in LN patients is accompanied by a decrease in Tregs, suggesting that Th17/Treg imbalance is involved in LN disease progression (64). *In vitro*, unstimulated bone marrow MSCs from BALB/c mice secrete TGF-β to induce Foxp3 expression to promote Treg cell development (66). As with TGF-β1, PGE2 secreted by human MSCs derived from bone marrow induces Foxp3+ Treg cell differentiation *in vitro* (67). Darlan et al. (59) showed that TGF-β1 released from human umbilical cord MSCs promotes the production of iTreg cells in peripheral blood mononuclear cells from lupus patients, characterized by Foxp3 expression. *In vitro* experiments showed that inhibition of CD4+ T cell differentiation to Th17 by MSCs derived from mouse bone marrow is triggered by cell-cell contacts and mediated by prostaglandin E2 (PGE2) *via* receptor 4 (EP4) (68). Transplantation of human umbilical cord MSCs into SLE patients decreased peripheral blood Th17 cells and elevated Treg cells, which were confirmed to be mediated by PGE2 and TGF-β secreted by MSCs in *in vitro* experiments (69). Makino et al. (70) showed that human supernumerary tooth-derived stem cells upregulate Tregs and downregulate Th17 activity and IL-17 in the kidney in MRL/lpr mice, while improving renal function, reducing collagen and IgG deposition in the glomerulus, and thinning the basement membrane. These studies indicate that MSCs directly upregulate Tregs or downregulate Th17 cells, thereby improving SLE or LN *via* the regulation of the Th17/Treg ratio.

## Immunomodulatory Activity of MSCs on Tfh Cells

Tfh cells are a specialized subset of CD4+ T cells that are located in B cell follicles. Studies have demonstrated that dysregulated Tfh cells are present in both SLE patients and animal models (71–74). Tfh cells can migrate into B cell follicles *via* CXCR5, leading to germinal center formation, promote the differentiation of B cells in the germinal centers into memory B cells or plasma cells (75). Studies showed that upon the existence of a defective checkpoint in the maintenance of peripheral B cell tolerance, which appears to be specific to patients with SLE, Tfh cells provide their cognate response B cells with the essential help needed to survive and pass the tolerance checkpoint (76, 77). Moreover, before B cells in the germinal center differentiate into memory B cells or plasma cells, Tfh cells signal B cells to undergo Ab class switching and affinity maturation (76). Studies have shown that in MRL/lpr mice, large numbers of Tfh cells are closely associated with excessive germinal center formation, high autoantibody titers, and damage from LN (78). IL-21 is a key cytokine produced by Tfh cells, which promotes the differentiation of Tfh. Bone marrow MSCs from C57BL/6 inhibit Tfh cell proliferation *in vivo* and *in vitro*, suppress Tfh differentiation by inhibiting IL-21, inhibit Tfh differentiation in the spleen of MRL/lpr mice, attenuate proteinuria and renal pathology, and prolong the survival of mice (79). Human umbilical cord MSCs inhibit Tfh proliferation by activating inducible NOS (iNOS) in lupus-prone B6.MRL-Faslpr mice, and improve the reduction of interstitial and peritubular inflammatory cell infiltration and reducing glomerular IgG deposition in mice (80). Human bone marrow MSCs can improve renal function and pathological alterations by inhibiting the emergence of Tfh in NZB/W mice, such as reduced proteinuria, decreased immune complex deposition, glomerular proliferation, and plasma cell infiltration, and *in vitro* human bone marrow MSCs directly inhibit the differentiation of nascent CD4+ T cells to Tfh cells in a contact-dependent manner (81). In summary, MSCs improve LN by inhibiting the proliferation and differentiation of Tfh cells.

## Immunomodulatory Activity of MSCs on DCs

DCs are the most functional and powerful antigen-presenting cells in the body, linking innate and adaptive immunity (82). Depending on the origin of DCs, they can be classified as plasmacytoid dendritic cells (pDCs), myeloid dendritic cells (mDCs), and peripheral blood monocyte-derived dendritic cells (PBMDCs), which accumulate in the kidney of LN patients and participate in the immune inflammatory response by expressing multiple receptors, co-stimulatory molecules, and inflammatory factors. pDCs are decreased in the peripheral blood and increased in the renal tissue of patients with active LN (83). pDCs secrete a large amount of type I interferons to trigger antiviral immune responses, and they have been identified as an important source of IFN-α production in SLE (84). Studies have shown that pDCs highly express IL-18R, which interacts with the cytokine IL-18 secreted by monocytes/macrophages to induce Th homeostasis to Th1 cell polarization (84). The main role of pDCs in SLE is to recognize nuclear autoantigens (85).pDCs promote the proliferation and cytotoxic effects of NKs and promote the renal inflammatory response (84). IFN-α is a type I IFN, and type I IFNs plays a key role in the pathogenesis of LN. IFN-α induces B cell differentiation and activation (86), upregulates BAFF (87, 88), promotes survival and maturation of DC cells (89), decreases Treg cells, and enhances Th cell functions (90). Elevated serum IFN-α levels in SLE patients correlate with disease activity and severity (91). Injection of IFN-α adenovirus rapidly induces T cell activation and extensive GC formation and produces large numbers of short-lived plasma cells producing IgG2a and IgG3 autoantibodies, leading to glomerulonephritis in lupus-prone NZB/W mice (87). mDCs are activated by IC and upregulate the expression of co-stimulatory molecules (CD86 and CD40), MHC molecules, and inflammatory factors (IL-6, TNF-α, etc.) to present antigens to T lymphocytes. Monocytes induced to differentiate into DCs can effectively present nuclear antigens, leading to cell proliferation, increasing secretion of inflammatory factors, and upregulated expression of co-stimulatory molecules, which play a vital part in maintaining a high level of an inflammatory environment. They have enhanced chemotactic properties that allow them to accumulate in secondary lymph nodes and inflammatory sites (92). CD1c+ DCs may be a key DC subtype for improving immune dysfunction and maintaining immune homeostasis. CD1c+ DCs were markedly reduced in SLE patients, especially LN patients, compared to that in healthy individuals (93).

MSCs can impair the antigen-presenting function of DCs by inhibiting their differentiation, maturation, and migration. MSCs have been shown to inhibit the differentiation of monocytes to DCs by reducing the expression of CD1-α, CD80, CD83, CD86, and HLA-DR on the surface of DCs (94, 95). MSCs inhibit DC cell maturation by secreting IL-6 (96). MSCs can also induce the production of regulatory DCs and evade apoptosis, further enhancing phagocytosis and inhibiting T cell activation and proliferation (97, 98). Bone marrow MSCs derived from C57BL/6 mice abolished the generation of functional IFN-α-producing pDCs, while they interfered with the maturation of mDCs (99). Significantly increased CD1c+ DCs and decreased proteinuria levels in SLE patients after human umbilical cord MSCT demonstrated that proliferation of tolerogenic CD1c+ DCs and inhibition of their apoptosis were promoted through IFNγ-FLT3L-FLT3 interactions (93). Human bone marrow-derived MSCs inhibit inflammatory factor IFN- α expression in Adriamycin (ADR)-induced nephropathy mice to suppress renal inflammation. In addition, MSCT prevents podocyte injury and renal fibrosis (100). In conclusion, MSCs can improve LN by inhibiting the differentiation and maturation of DCs and by suppressing the production of their cytokine, IFN-α.

## Immunomodulatory Activity of MSCs on Macrophages

Macrophages are a prevalent infiltrative population in the kidney of LN patients, and studies have shown that renal macrophage infiltration is related to poor prognosis, and that macrophage depletion improves LN, suggesting a key role for macrophages in LN (101). Simultaneously, activated macrophages may be involved in LN through TNF-α and IL-1β-mediated podocyte injury (102, 103). Recently, new concepts of macrophage subsets have emerged, including the classically activated pro-inflammatory M1 type and the alternative activated anti-inflammatory M2 type (104). CD206 is an important marker of M2 type macrophages, and macrophage CD206 is expressed at low levels in B6.MRL-Fas^lpr^ mice and SLE patients (105). The balance between M1 and M2 macrophages is associated with the pathogenesis of nephritis, and dysregulated M2 macrophages play a pro-inflammatory part in LN (106). Iwata et al. (107) showed that in lupus-susceptible MRL/lpr mice, abnormal macrophages fail to shift from a “destructive” inflammatory phenotype to a “healing” anti-inflammatory phenotype, which triggers LN. Furthermore, certain cytokines produced by macrophages such as IL-10 (108, 109), IL-12 (51), and TNF-α (110, 111) play important roles in LN. Human placental MSCs shift macrophage differentiation from M1 into M2 macrophages, helping to aid in the regression of inflammation and further tissue regeneration (112). Murine MSCs induce macrophage M2 polarization through secreted TGF-β to exert anti-inflammatory effects and enhance phagocytosis *via* the Akt/FoxO1 pathway (113). Human umbilical cord MSCs increase CD206 expression in lupus-prone mice and SLE patients to promote M2 type macrophages and their phagocytosis, and improve inflammatory response and renal injury (105). Zhang et al. (103) demonstrated that human umbilical cord MSCT into B6.lpr mice polarize macrophages to the anti-inflammatory M2 type while reducing macrophage infiltration in the kidney of B6.lpr mice to prevent podocyte injury and improve LN; examples include reduction of proteinuria and glomerular thylakoid cell proliferation, and thylakoid matrix deposition. Thus, MSCs improve LN by promoting the M2-type polarization of macrophages.

## Immunomodulatory Activity of MSCs on NKs

NKs are large granular lymphocytes which act as a bridge between the innate and adaptive immune systems. NKs also produce a variety of cytokines and chemokines, for instance TNF-α, CCL3, and CCL4, which amplify and recruit inflammatory responses through various mechanisms involved in the LN disease process (114, 115). Several studies have shown that the proportion of NKs and the total number of NKs in the blood of SLE patients are significantly lower, especially in LN patients; also, defective killing activity of NKs has been shown in the peripheral blood of first-degree relatives of SLE patients (116–118). Studies have shown that renal NKs from mice with active disease produced more cytotoxic granules (perforin and granzyme B) and IFN-γ when stimulated with PMA+ ionomycin, which contributed in part to the renal damage in SLE (119, 120). Human bone marrow MSCs inhibit IL-12-induced proliferation of NKs through the derived immunomodulatory factors IDO and PGE2, and MSCs inhibit cytotoxic activity and their cytokine IFN-γ production (121, 122). When MSCs are exposed to exogenous IFN-γ, the expression of MHC-I molecules is upregulated and NKs are insensitive to the killing ability of MSCs (123). NKs can kill autologous or allogeneic MSCs when activated by IL-12 and IL-15 (124, 125). In short, MSCs may improve LN by inhibiting the proliferation and activity of NKs; however, in an inflammatory environment, NKs may have the potential to lyse MSCs and affect their immuno-modulatory function.

## Immunomodulatory Activity of MSCs on Cytokines in LN

Large amounts of inflammatory mediators are produced in the LN kidney, and as the disease progresses, the response spreads (126). It is widely believed that ICs trigger the production of key mediators of inflammation, including cytokines, chemokines, and adhesion molecules, leading to glomerular infiltration of individual nucleated cells, tissue damage, and renal failure in mouse models of LN, and their absence or inhibition greatly diminishes disease activity. MSCs can exert immune activity by regulating the secretion of certain cytokines, for example IL-10, IL-17, TGF-β, TNF-α, GM-CSF, and HMGB-1 (**Table 1**).

**Table 1:** The role of cytokines in the pathogenesis of LN and the role of MSCs in the treatment of LN discussed in the text.

Cytokine type		Mechanism of action in LN	Synergistic cytokines	MSC function	References
IL	IL-10	Early stage: immunosuppression	IFN-γ	Promotion of secretion	(37, 108, 109, 127, 128, 127, 167)
		Late stage: promote B cells proliferation and differentiation, immunostimulatory effect	TNF-α	Suppression of secretion	
	IL-17	Induce production of inflammatory factors and recruitment to inflamed organs	ICAM-1	Suppression of secretion	(129–136)
	IL-12	Promote Th cell differentiation to Th1	IL-18, IFN-γ	Suppression of secretion	(51, 137–141)
	IL-6	Promote plasma cell proliferation, increases IgG secretion, and induces T and monocyte differentiation		Suppression of secretion	(142–150)
TNF	TNF-α	Early stage: induction of immune tolerance, immunosuppression			(140, 151–155)
		Late stage: promotes inflammation	IL-1 β、IL-6	Suppression of secretion	
	BAFF	Promotes B cells development		Suppression of secretion	(30–33, 37)
IFN	IFN-α	Upregulation of BAFF, promote DCs maturation, reduce Treg cells and enhance effector Th cells		Suppression of secretion	(87–91, 99, 100)
	IFN-γ	Promote macrophage and B cells activation and Th cells differentiation to Th1 cells		Suppression of secretion	(156–164)
HMGB-1	HMGB-1	Promotes activation of DCs, B cells, and cytokine secretion	TNF-α、IL-1	Suppression of secretion	(165, 166)

## Immunomodulatory Activity of MSCs on IL-10

IL-10 is a regulatory cytokine produced by B cells or certain CD+ 4 T cells and may have multiple roles in lupus, playing a major part in the stability of cellular and humoral immunity, with immunostimulatory and suppressive effects. Studies have indicated high levels of IL-10 in SLE patients and mouse models of lupus (108). IL-10 promotes B cell proliferation and differentiation (109), and continuous administration of anti-interleukin 10 antibody delays autoimmunity in NZB/W F1 mice by upregulating TNF-α (167). *In vitro* experiments show that bone marrow MSCs from BALB/c mice can reduce IL-10 secretion (37). However, genetic defects in IL-10 lead to more severe glomerulonephritis in mice with lupus in the MRL background, and IL-10 downregulates IFN-γ in the early stages of lupus to inhibit the pathogenicity of Th1 cells and delay the progression of lupus (127). Choi et al. (128) demonstrated that human adipose tissue-derived MSCs significantly increased the expression levels of IL-10 and IL-4 cytokines in the serum of (NZB × NZW) F1 mice and that they had decreased serum urea nitrogen levels and increased survival. In summary, MSCs improve LN by regulating IL-10 expression at different stages of LN.

## Immunomodulatory Activity of MSCs on IL-17

IL-17 is mainly produced by Th17 cells and other T cell subsets and plays a central part in the pathogenesis of LN. IL-17 induces the production of more inflammatory cytokines and chemokines, and promotes the recruitment of inflammatory cells (monocytes and neutrophils) to inflammatory organs (129, 130). IL-17 promotes T cell infiltration in tissues by stimulating the expression of intercellular adhesion molecule (ICAM)-1 (131) and promotes autoantibody production and renal inflammation in SLE (132). IL-17 plays a vital part in the pathogenesis of SLE and presents at high levels in the serum of SLE patients and lupus mice (133). IL-17 was detected in damaged renal tissues of SLE patients, and IL-17 in urine sediment was associated with LN activity (134). Sun et al. (135) showed that mouse bone marrow MSCs inhibited IL-17 in the bone marrow and spleen of MRL/lpr mice, improved kidney function, reduced circulating immunoglobulins, IgG and IgM, and restored glomerular structure. Adipose MSCs derived from C57BL/6 mice reduced IL-17 expression in the serum of MRL/lpr mice and IL-17 mRNA expression in renal tissues by inhibiting the mTORC1 pathway, improving renal function, reducing glomerular tract proliferation, inflammatory cell infiltration, and C3 deposition (136). Therefore, MSCs prevent and ameliorate LN by downregulating the expression of IL-17.

## Immunomodulatory Activity of MSCs on IL-12

IL-12 is a 70 kDa heterodimer (IL-12p70) produced by macrophages and DCs, and together with IL-18, it promotes the production of IFN-γ and is involved in the differentiation and activation of various Th cell subpopulations (51, 137). Tucci et al. (51) demonstrated that serum IL-12 levels are higher in SLE patients; IL-12 triggers an inflammatory response in the kidney and promotes the conversion of Th cells to Th1, and that IL-12 responds to the severity of LN. IL-12 is upregulated in the kidney and serum of MRL-Fas^lpr^ mice with LN and its expression further increases with disease progression (138, 139). Human umbilical cord blood MSCT reduces NZB/W F1 mouse IL-12 to inhibit the inflammatory response, reduces the severity of proteinuria, the progression of renal function deterioration, and reduces glomerular tract proliferation and sclerosis (140). Human adipose-derived MSCs reduce serum IL-12 levels in MRL/lpr mice and decrease inflammatory cell infiltration, IgG and C3 deposition in the kidney (141).

## Immunomodulatory Activity of MSCs on IL-6

IL-6 is a multipotent cytokine. IL-6 promotes plasma cell survival and proliferation (142), enhances IgG secretion (143), induces differentiation of T cells into effector cells (144), and induces differentiation of monocytes into macrophages (145, 146). IL-6 is significantly elevated in the urine of LN patients, may be associated with LN activity (147, 148), and has emerged as a potential biomarker, with IL-6 deficiency reducing macrophages, CD4+ and CD8+ lymphocytes, along with reduced renal IgG and C3 deposition and delayed LN in MRL-Fas^lpr^ mice (149). Thiel et al. (150) showed that human embryonic stem cell-derived MSCs reduce serum IL-6 levels in BWF1 mice and have the ability to prevent or slow down lupus-associated glomerular disease.

## Immunomodulatory Activity of MSCs on TNF-α

TNF-α is a multipotent cytokine, mainly produced by activated macrophages and lymphocytes, that exerts pro-inflammatory and immunomodulatory effects in LN. TNF-α has an immunosuppressive effect at the onset of LN, and administration of TNF-α to pre-onset NZB/W mice delayed the onset of LN; TNF-α may exert a protective effect by inducing tolerance (151). However, TNF-α causes end-organ damage in late disease (151). Studies have demonstrated a major involvement in the inflammatory cascade response in kidney injury, such as the promotion of IL-1 β and IL-6 secretion mediated by dsDNA antibodies (152), elevated TNF-α levels in the kidney in LN, and correlation with disease activity (153, 154). Chang et al. (140) showed experimentally that human umbilical cord blood MSCT could inhibit the production of the pro-inflammatory factor TNF-α and improve LN. Liu et al. (155) showed that human placenta-derived MSCT downregulated the expression of the inflammatory marker TNF-α in the kidney and improved renal function and pathology, such as reducing proteinuria, glomerular inflammatory infiltrates, and immune complex deposition.

## Immunomodulatory Activity of MSCs on IFN-γ

IFN-γ is a type II IFN, mainly produced by Th1 and NKs. IFN-γ promotes B cell activation and production of IgG2a, IgA, and IgM (156, 157). IFN-γ mediates the activation of macrophages (158), induces differentiation of Th cells to Th1 cells, and inhibits the proliferation of Th2 cells (159, 160). Glomerulonephritis is severely reduced in IFN-gamma-/- mice and overproduction of IFN-γ is required for the development of lupus (161). Jacob et al. (162) showed that treatment with IFN-γ accelerated the development of nephritis in (NZB × NZW) F1 mice and that application of a specific IFN-γ monoclonal antibody delayed the development of LN. Ozmen et al. (163) showed that treatment of NZB/W mice with soluble IFN-γ receptors delayed the development of glomerulonephritis. Significantly lower levels of IFN-γ were found in T lymphocytes from SLE patients co-cultured with human umbilical cord-derived MSCs (164).

## Immunomodulatory Activity of MSCs on HMGB-1

HMGB-1 is an immunostimulatory cytokine mediator that mediates IC to stimulate DC and B cell activation and production of cytokines, such as TNF-α and IL-1, and plays a main part in the development of LN (165). Gu et al. (166) found that mRNA expression of HMGB-1 was reduced in the umbilical cord MSC group compared with the control group by transplanting human umbilical cord MSCs in MRL/lpr mice; the rate of positive immunohistochemical staining for HMGB-1 was lower than that of the control group, also improved kidney function and crescent reduction was demonstrated.

## Failure of MSC Therapy and Analysis of Causes

**Table 2** demonstrates several preclinical studies of MSCs in lupus-susceptible mice, which have been extensively conducted and yielded preliminary results, and these results have facilitated current clinical studies. Many *in vivo* studies have shown differences in the immunomodulatory activity of MSCs in LN, and the previous section of this review described the immunomodulatory activity and potential mechanisms of MSCs in LN, which support the preclinical and clinical applications of MSCs; however, the applications of MSCs in regenerative medicine are still challenging; hence, this section focuses on elucidating the contradictory findings of preclinical and clinical studies and analyzing the reasons for the conflicting results.

**Table 2:** Preclinical study of MSCs in LN.

MSC types	Experimental models	Injection methods	Dose and Frequency	Reference
Bone marrow MSC-derived from Balb/c mice	MRL/lpr mice	intravenous	1× 10^6^MSCs, at 18 weeks of age	Ma et al. (37)
Human adipose-derived MSCs	Roquin san/san C57BL/6 mice	tail vein	1×10 ^6^ MSCs, at age 17 weeks, once weekly for 5 weeks	Park et al. (38)
Human gingiva-derived MSCs	NZM2838 mice	intravenous	2 × 10 ^6^ MSCs, at 10 or 20 weeks of age	Dang et al. (39)
Human umbilical cord-derived MSCs	B6.MRL-Fas^lpr^ (B6.lpr) mice	tail vein	1 × 10^6^ MSCs, at twenty-eight-week old	Huang et al. (49)
Human supernumerary tooth-derived stem cells	MRL/lpr mice	intravenous	0.1 x 10^6^/10 g body weight, at the age of 16 weeks	Makino et al. (70)
C57BL/6 (B6) mice bone marrow MSCs	MRL/lpr mice	tail vein	2× 10^6^ MSCs, twice (at the age of 18 and 20 weeks)	Yang et al. (79)
Human Umbilical Cord MSCs	B6.lpr mice	tail vein	1 × 10^6^ MSCs, at the age of 6 months	Zhang et al. (80)
Human bone marrow MSCs	NZB/W mice	retro-orbital injection	1 × 10^6^ MSCs, at 17, 19, and 21 weeks of age	Jang et al. (81)
Human Placenta-Derived Mesenchymal Stem Cells	MRL/lpr mice	tail vein	1 × 10 ^6^MSCs, at the 16th, 18th, and 20th week of age	Liu et al. (155)
Human adipose tissue-derived MSCs	C3.MRL-Fas^lpr^/J mice	intravenous	1 × 10 ^6^MSCs, every two weeks at 5 weeks a total of 18 times	Choi et al. (55)
Human adipose tissue–derived mesenchymal stem cell	(NZB × NZW)F 1 mice	intravenous	1× 10^6^ MSCs, every 2 weeks from age 5 weeks until age 23 weeks	Choi et al. (128)
Mice adipose-derived MSCs	MRL/lpr mice	intravenous	2×10^5^/10g MSCs, from age 28 to 31 weeks, for a total of four injections	Wei et al. (136)
Human embryonic stem cell-derived MSCs	NZBxNZW F1 (BWF1)	intravenous	5 × 10^5^ MSCs, from 23–33 weeks of age in a bi-weekly	Thiel et al. (150)
Bone marrow MSCs from BALB/c mice	NZB/W mice	intraperitoneal injection	1× 10^6^ MSCs, at 21 or 32 weeks of age	Youd et al. (168)
Bone marrow MSCs from MRL/lpr mice	MRL/lpr mice	intravenous	0.1 × 10^6^/10 g MSCs, at 18 weeks of age	Che et al. (17)
Bone marrow MSCs derived from C57BL/6J	NZB/Wf1 mice	tail Vein	1×10^6^ MSCs/kg, at 18 or 22 weeks of age	Tani et al. (169)

Most MSCTs have been shown to improve LN, including improving renal function such as reducing proteinuria, serum urea nitrogen, and creatinine levels, and improving renal pathology such as reducing glomerular tract proliferation, inflammatory cell infiltration, and immune complex deposition. However, there are contradictory results, as Youd et al. showed in a pilot study that bone marrow MSCs from BALB/c mice injected intraperitoneally into NZB/W mice showed that MSCT-treated mice exacerbated the disease instead of benefiting from it (168), probably due to the intraperitoneal injection method, in which MSCs injected intraperitoneally into mice could form aggregates with B20 aggregates, lymphocytes, and macrophages, which then attach to the peritoneal wall, thus limiting the entry of MSCs into the body circulation (170). Che et al. (17) also found that transplantation of bone marrow MSCs derived from MRL/lpr mice into MRL/lpr mice did not improve renal immune complex deposition and had no significant inhibitory effect on B lymphocytes, due to the defective immune function of bone marrow MSCs derived from lupus mice. Tani et al. (169) showed that transplantation of bone marrow MSCs derived from C57BL/6J-derived bone marrow MSCs transplanted into NZB/Wf1 mice showed only delayed onset of proteinuria, no improvement in anti-ds-DNA titers and renal function scores, more inflammatory cell deposition, B cell overexpression and fewer Treg cells. This may be due to different murine models of lupus and the multiplicity and heterogeneity of MSCs, among other reasons.

Clinical cases of MSC-treated LN have accumulated through a large number of preclinical trials. Most clinical trials used an intravenous injection of 1 × 10^6^ MSCs/kg, with good results in LN patients. Wang et al. (8) observed the clinical performance of 40 SLE patients treated with human Umbilical Cord-MSCs. After 12 months of follow-up, the overall survival rate was 92.5%. In the UC-MSC group, SLEDAI scores decreased, as also the levels of serum creatinine, urea nitrogen, and proteinuria. Gu et al. (171) showed that 81 patients with refractory or active LN treated who had undergone allogeneic bone marrow MSCT for LN had an overall survival rate of 95% at 12-month follow-up. Patient survival had improved, and SLEDAI scores, serum creatinine, and urine protein levels had decreased. The observation of 87 patients with refractory SLE and receiving intravenous human bone marrow/umbilical cord MSCs demonstrated an overall survival rate of 94%, an improved renal function, and the recovery of serum albumin and complement C3 levels during 4 years of follow-up, suggesting that allogeneic MSCT can lead to clinical remission and improvement of renal dysfunction in patients with drug resistant SLE (172). However, there are still a few reports of adverse events associated with MSC transplantation. The disease activity of two SLE patients failed to improve after autologous MSCT, and the observed increase in Treg cells was not associated with clinical benefits, mainly due to the possible presence of defective MSCs in lupus patients (9). Deng et al. (173) used a randomized two-way blinded approach to divide patients into a human umbilical Cord-MSC group and a placebo group. There were two adverse events in each group: stroke and ascites in the placebo group, and leukopenia and pneumonia in the human umbilical Cord-MSC group, with one patient dying of pneumonia. Allogeneic normal MSCT is more effective than autologous MSCT in treating SLE (9, 174).

In SLE, the body is in a chronic inflammatory state in which MSCs can act to promote or suppress the immune system. High levels of TGF-β production by umbilical cord MSCs, upregulation of Treg cells and PGE2, and downregulation of Th17 cells are responses to stimulation of the lupus microenvironment to induce immune tolerance (69). Overexpression of IL-37 may enhance the immunosuppressive effect of MSCs by suppressing the inflammatory microenvironment, while transplanted MSCs may improve the damaged microenvironment in SLE (175). However, high levels of TNF-α in SLE patients significantly inhibit the migration and homing ability of bone marrow MSCs (18). The inflammatory environment promotes the secretion of inflammatory cytokines such as IL-1β and TNF-α by MSCs, and in response to the co-stimulation of IL-1β and TNF-α, MSCs promote inflammatory responses and Th17 differentiation and participate in the progression of SLE disease (176). MSCs with SLE are more susceptible to the effects of the matrix proteins of the bone marrow microenvironment and cytokines and chemokines secreted by stromal cells during culture, which accelerate senescence (10). The immunomodulatory effects of MSCs are influenced by the tissue microenvironment, and modification of MSCs with cytokines or drugs to enhance their therapeutic potential requires further exploration.

## Conclusion and Outlook

With the development and application of stem cell technology, the immunomodulatory activity of stem cells can be utilized to improve LN, which has greatly contributed to the development of LN treatment. We illustrate the defective MSCs in SLE and describe the pathogenesis of LN in detail in terms of immune cells and cytokines and discussed the immunomodulatory activity and potential mechanisms of MSCs in LN, all of which support the preclinical and clinical applications of MSCs. Although MSCs are promising in the treatment of LN, the application of MSCs in regenerative medicine remains challenging, as there are many differences in the immunomodulatory activity of MSCs due to multiple factors, for example, differences in the source of MSCs, injection routes, and the amount of injected dose. Also, the immunomodulatory effect of MSCs may be influenced by the layout microenvironment, so there is a need to find standardized ways or pretreatment of MSCs to avoid these situations. In the future, we will fully exploit the immunomodulatory activity of MSCs and use it to optimize their therapeutic effects. We are optimistic that MSCs will be valued as immunomodulators of autoimmune responses, as there is ample proof of principle for conducting preclinical and clinical experiments.

## Author Contributions

JL: conceptualization and writing – original draft. ML: writing – review and editing. BL: visualization. YL: writing – review and editing. YZ: writing – review and editing. XB: writing – review and editing. BS: visualization. XL: review and project administration. PL: funding acquisition, and supervision. All authors contributed to the article and approved the submitted version.

## Funding

This work was supported by the National Natural Science Foundation of China [grant number 81970628(PL)] and the Science and Technology Development Plan Project of Jilin province [grant numbers 20200201488JC(BL) and 20190304042YY(PL)].

## Conflict of Interest

The authors declare that the research was conducted in the absence of any commercial or financial relationships that could be construed as a potential conflict of interest.

## Publisher’s Note

All claims expressed in this article are solely those of the authors and do not necessarily represent those of their affiliated organizations, or those of the publisher, the editors and the reviewers. Any product that may be evaluated in this article, or claim that may be made by its manufacturer, is not guaranteed or endorsed by the publisher.

## Abbreviations

ADR, Adriamycin; Anti-dsDNA, anti-double-stranded DNA; Breg, Regulatory B cells; BAFF, B cell activating factor; CCL2, Chemokine ligand 2; CXCR4/CXCR5, chemokine receptor 4/5; DCs, Dendritic cells; ERS, Endoplasmic reticulum stress response; EP4, receptor 4; FOXP3, Forkhead box P3; HGF, Hepatocyte growth factor; IC, Immune complexes; ICAM, Intercellular adhesion molecule; IDO, Indoleamine 2,3dioxygenase; IL, Interleukin; INF, Interferon; LN, Lupus nephritis; iTreg, inducible Treg; iNOS, inducible NOS; mDCs, Myeloid dendritic cells; MSCs, Mesenchymal stem cells; MSCT, Mesenchymal stem cell transplantation; MAVS, Mtochondrial antiviral signaling; NKs, Natural killer cells; PBMDCs, Peripheral blood monocyte-derived dendritic cells; pDCs, Plasmacytoid dendritic cells; PGE2, prostaglandin E2; ROS, Reactive oxygen species; SA-b-gal, Senescence associated b-galactosidase; SLE, Systemic lupus erythematosus; STAT3, Signal transducer and activator of transcription 3; TGF- b, Transforming growth factor b; Th, T helper cells; Tfh, Follicular helper T cells; TNF, Tumor necrosis factor; Treg, Regulatory T cells.
